# Ruptured Wilms Tumor: Clinical Features, Diagnostic Challenges, and Survival Outcomes

**DOI:** 10.3390/curroncol33050293

**Published:** 2026-05-19

**Authors:** Hiba Emadeldeen, Khalil Ghandour, Tamador Al-Shamaileh, Ahmad Kh. Ibrahimi, Nasim Sarhan, Iyad Sultan, Hadeel Halalsheh

**Affiliations:** 1 Department of Pediatrics, King Hussein Cancer Center, Amman 11941, Jordanisultan@khcc.jo (I.S.); 2 Department of Pediatric Surgery, King Hussein Cancer Center, Amman 11941, Jordan; jafapal48@yahoo.com (K.G.);; 3 Department of Radiation Oncology, King Hussein Cancer Center, Amman 11941, Jordan; 4 Artificial Intelligence Office, King Hussein Cancer Center, Amman 11941, Jordan; 5 Department of Pediatrics, The University of Jordan, Amman 11941, Jordan

**Keywords:** Wilms tumor, nephroblastoma, tumor rupture, masking effect, SIOP, survival, Middle East

## Abstract

Background: Wilms tumor (WT) rupture is a serious complication that upstages the disease and requires treatment intensification. This study evaluates clinical characteristics, radiological-pathological concordance, and survival outcomes of ruptured versus non-ruptured WT at a major Middle Eastern tertiary center. Methods: We conducted a retrospective cohort study of 111 pediatric patients with unilateral WT treated at King Hussein Cancer Center, Jordan, between October 2014 and December 2023 (follow-up to December 2025). Tumor rupture was defined by preoperative CT findings (peritumoral effusion, hemorrhage, or peritoneal nodules), intraoperative capsular breach/spillage, or pathological confirmation. Event-free survival (EFS) and overall survival (OS) were estimated using Kaplan–Meier methods and compared with the log-rank test. Multivariable Cox regression identified independent prognostic factors. Results: Tumor rupture occurred in 17 patients (15.3%). Ruptured cases were older (median 4.2 vs. 3.5 years, *p* = 0.03), had larger tumors (13.7 vs. 11.7 cm, *p* = 0.01), and presented with lower hemoglobin (7.9 vs. 10.4 g/dL, *p* < 0.001). All ruptured cases were stage III/IV, with 41% having distant metastases at diagnosis. Five-year EFS was 44.1% vs. 75.8% (*p* = 0.025) and OS was 58.2% vs. 81.4% (*p* = 0.002) for ruptured vs. non-ruptured groups. On multivariable analysis, rupture independently predicted death (HR 17.62, 95% CI 2.69–115.48, *p* = 0.003) and relapse (HR 8.1, 95% CI 1.66–39.57, *p* = 0.01). Conclusion: WT rupture is associated with advanced disease at presentation and significantly inferior survival. Substantial discordance between preoperative radiological/intraoperative findings and post-chemotherapy pathology highlights the “masking effect” of neoadjuvant chemotherapy. A multidisciplinary approach integrating initial imaging, surgical notes, and histology is essential to avoid undertreatment in SIOP-based protocols.

## Simple Summary

This retrospective study demonstrates that Wilms tumor rupture significantly reduces survival and identifies a 58.8% “masking rate” where neoadjuvant chemotherapy obscures rupture on pathology, necessitating a multidisciplinary “highest-stage” approach to prevent undertreatment.

## 1. Introduction

Wilms tumor (WT) is the most common pediatric renal malignancy, accounting for approximately 6% of all childhood cancers [1,2,3]. Modern multimodal therapy has achieved overall survival (OS) rates exceeding 90% in high-income countries [1]. However, tumor rupture, which may occur spontaneously or intraoperatively during tumor resection, remains a major challenge; the reported incidence ranges between 2% and 10% and carries significant clinical implications [4,5].

Rupture occurs due to capsule disruption. It is either contained retroperitoneally or the tumor spills freely into the peritoneal cavity, thus increasing the risk of loco-regional relapse [5,6,7]. Spontaneous rupture is typically associated with large tumors, particularly among those exhibiting necrosis or hemorrhage [5,7,8,9]. Clinically, it may present with acute abdominal distension, pain, pallor, vomiting, and, in severe cases, hypovolemic shock. Laboratory evaluations often reveal a drop in hemoglobin levels due to intra-abdominal bleeding [6,8].

Under the SIOP approach, which uses neoadjuvant chemotherapy, accurate identification of preoperative rupture is complicated by the potential “masking effect,” whereby chemotherapy can obscure prior capsular breaches on final pathology. Preoperative radiological signs suggestive of tumor rupture include contained peritumoral or intraperitoneal fluid collections, irregular tumor margins, capsular disruption, and evidence of hemorrhage. Computed tomography (CT) imaging may further demonstrate hemoperitoneum or peritoneal nodularity, indicating possible tumor spread [5,6,10]. Intraoperative rupture usually occurs during tumor resection, particularly when the tumor is large, friable, or adherent to surrounding tissues [7,10,11].

Tumor rupture is a well-established adverse prognostic factor, necessitating intensive therapy. Most guidelines recommend upstaging to stage III with intensified chemotherapy (addition of doxorubicin) and abdominal radiotherapy [12,13,14,15]. While this intensive therapy aims at reducing the risk of recurrence, it increases the treatment burden and is associated with long-term toxicities, including cardiotoxicity, infertility, and secondary malignancies [8,10,16,17].

The prognostic significance of tumor rupture is inconsistent across studies; whereas some reports describe worse event-free and overall survival compared with patients without rupture, others have found no significant difference in outcomes [5,10,18].

This study describes the clinical features, diagnostic challenges, and survival impact of ruptured WT in a Middle Eastern tertiary center and emphasizes the importance of multimodality assessment.

## 2. Patients and Methods

Following the study approval from King Hussein Cancer Center (KHCC) Institutional Review Board (IRB-25KHCC076), a retrospective medical record review was conducted at KHCC, Jordan. The study included pediatric patients diagnosed with unilateral WT between October 2014 and December 2023, with follow-up data available through December 2025. Eligible patients were identified through institutional records. We included patients with complete medical records, including imaging, surgical, pathological, and treatment details. Patients who received any part of their treatment outside KHCC or were referred after prior treatment elsewhere were excluded. We excluded patients with bilateral disease due to the complexity of treatment.

Data were collected from both electronic and paper medical records, and included patients’ demographics (age, sex), clinical presentation (symptoms, hemoglobin level), imaging features (laterality of the tumor, tumor size, radiologic signs of rupture), tumor characteristics (histologic subtype, staging), treatment modalities (surgery, chemotherapy regimen, radiotherapy use), and clinical outcomes.

Tumor rupture was defined by any of the following: (1) Radiological: preoperative contrast-enhanced CT showing peritumoral or peritoneal hemorrhage/effusion or peritoneal nodules. (2) Intraoperative: surgeon-documented capsular breach or tumor spillage in operative notes. (3) Pathological: histological evidence of capsular rupture. Patients were categorized into two groups: those with tumor rupture and those without.

Patients received treatment based on International Society of Pediatric Oncology (SIOP) protocols. Between 2014 and 2016, the SIOP-2001 protocol was followed, while from 2017 onward, the SIOP-UMBRELLA protocol was implemented [15].

Preoperative chemotherapy consisted of vincristine and dactinomycin (AV) for localized disease (4 weeks) or the addition of doxorubicin (AVD) for metastatic cases (6 weeks). Post-operative chemotherapy was administered according to a risk-adapted protocol, with regimens stratified by histological risk group, surgical stage (I–III), and tumor volume following neoadjuvant chemotherapy. Patients with low-risk Stage I tumors required no further systemic therapy, while those with Stage II or III disease received the AV2 regimen. For intermediate-risk tumors, treatment intensity escalated based on surgical stage and post-neoadjuvant tumor volume (≤500 mL versus >500 mL), utilizing AV1, AV2, or AVD regimens. Patients with high-risk histologies, including blastemal type and diffuse anaplasia, received intensified systemic therapy regardless of tumor volume, utilizing the AVD regimen for Stage I and the multi-agent high-risk (HR-1) regimen for Stages II and III.

Radiotherapy Protocol Summary: Post-operative RT was administered based on histological risk and surgical staging, typically delivered within 2–4 weeks post-surgery. Local flank RT was indicated for Intermediate Risk Stage III disease and High Risk Stage II/III disease (excluding Stage II blastemal subtype). Standard doses for the flank ranged from 14.4 Gy for Intermediate Risk to 25.2 Gy for High Risk histologies. Whole-abdomen RT was reserved for cases of diffuse intra-abdominal spread or major rupture, with doses of 15.0 Gy (Intermediate Risk) or 19.5 Gy (High Risk). For patients under 2 years of age, total doses and fraction sizes were reduced to mitigate toxicity. Treatment planning utilized preoperative contrast-enhanced CT or MRI to delineate the Clinical Target Volume, defined as the preoperative Gross Tumor Volume plus a 1 cm margin. The Planning Target Volume included an additional 1 cm margin for movement. Either Three-Dimensional Conformal Radiation Therapy (3D-CRT) or Intensity-Modulated Radiation Therapy (IMRT) technique was used, depending on the era of treatment. Macroscopic residual disease received an additional 10.8 Gy boost.

Every case was thoroughly reviewed by the multidisciplinary team (MDT) at initial presentation and at the time of local control. The tumor rupture management plan was discussed at initial presentation, local control planning, and once the pathology report became available. The MDT is a team that includes a pediatric oncologist, pediatric surgeon, radiologist, and radiation oncologist, thus ensuring comprehensive discussions for each case.

### Statistical Analysis

Demographic, tumor, and treatment characteristics were summarized by descriptive analysis. Categorical variables were compared using Pearson’s Chi-squared or Fisher’s exact tests, while continuous variables were analyzed using the Wilcoxon rank-sum test. OS was defined as the time between diagnosis and death from any cause or the last follow-up for patients remaining alive. EFS was defined as the time between diagnosis and the occurrence of disease recurrence or progression, second malignancy, death, or last follow-up for patients who did not experience an event. OS and EFS were calculated using the Kaplan–Meier method. The log-rank test was used to compare survival curves when needed. Cox proportional hazards regression was performed for univariable and multivariable analyses to identify factors associated with EFS and OS. Hazard ratios (HRs) with 95% confidence intervals (CIs) and *p*-values were reported. A *p*-value of 0.05 or less was considered statistically significant.

## 3. Results

### 3.1. Patients and Clinical Characteristics

During the study period, 111 pediatric patients were diagnosed with unilateral WT. A slight female predominance was noted (57%) with a median age of 3.8 years (range, 0.4–15.1). Abdominal mass was obvious in 83 patients (75%), while abdominal pain (*n* = 38, 35%) and hematuria (*n* = 23, 21%) were present. Metastasis at diagnosis was present in 35 patients (32%), and 17 (15.3%) had tumor rupture. Patients’ characteristics are summarized in Table 1.

### 3.2. Characteristics of Patients with Ruptured WT

Of the 17 patients with tumor rupture, the vast majority (*n* = 15, 88.2%) presented with preoperative rupture identified on initial imaging. Intraoperative rupture occurred in only two patients (11.8%). The median age at diagnosis was 4.2 years (range: 2.6 to 15.1 years), and 11 patients (65%) were female. Metastatic disease was present in seven patients, and six had unfavorable histology (four with diffuse anaplasia and two with blastemal-predominant tumors). Abdominal pain was reported in eight patients (47%), and twelve patients (70%) had hemoglobin levels below 9 g/dL, Table 1.

### 3.3. Diagnostic Discordance and the “Masking Effect”

CT imaging suggestive of preoperative tumor rupture included retroperitoneal peritumoral effusion or hemorrhage in seven patients (46.7%), peritoneal hemorrhage or peritoneal nodules in six patients (40.0%), and bloody ascites in seven patients (46.7%).

Intraoperative confirmation of rupture in five patients. In seven additional cases, the tumor was found to be densely adherent to adjacent structures such as the liver, diaphragm, or bowel, raising concern for preoperative rupture. No intraoperative evidence of rupture was observed in the remaining three patients.

Final pathology confirmed capsular rupture in only six patients (35.3%). In 10 patients (58.8%), the capsule was reported as intact on pathology despite radiological or intraoperative evidence of rupture in several cases. Among these, intraoperative rupture was documented in three cases, radiological evidence of bloody ascites in two, and peritoneal nodules with hemorrhage in two additional patients.

In two patients, capsular breach and tumor spill occurred during tumor resection: in one case, during upfront resection elsewhere. The second patient developed intra-tumoral hemorrhage after two cycles of neoadjuvant chemotherapy, resulting in respiratory distress and hemodynamic instability that necessitated urgent surgical intervention. The spill most likely occurred due to the large tumor size (17.5 cm) and fragile capsule.

### 3.4. Comparison of Patients With and Without Rupture

Patients with rupture were significantly older at diagnosis compared to those without rupture (median age: 4.2 vs. 3.5 years, *p* = 0.03). Rupture was strongly associated with advanced disease stage; 41% of patients with rupture had stage IV disease compared to only 30% in the non-ruptured group (*p* < 0.001). Additionally, ruptured tumors were significantly larger (median size: 13.7 cm vs. 11.7 cm, *p* = 0.01) and were associated with lower hemoglobin levels at presentation (7.9 g/dL vs. 10.4 g/dL, *p* < 0.001). Details of patients’ characteristics are in Table 1.

Treatment regimens were more intensive among patients with tumor rupture. Doxorubicin-based chemotherapy was administered to 94% of patients in the ruptured group compared to 45% in the non-ruptured group (*p* < 0.001). Whole-abdomen radiotherapy was also more frequently used in the rupture group (35% vs. 0%, *p* < 0.001). In the non-ruptured group, 30.9% received flank radiotherapy, which was necessitated by Stage III disease indications other than rupture (positive lymph node involvement or microscopic residual disease) (*n* = 23) or unfavorable histology in stage I and II (*n* = 6). Histological subtypes were comparable between the two groups, with favorable histology predominating.

After a median follow-up of 21.1 months (range, 5.6–109.6 months) for the rupture group, eight patients experienced disease-related events, including progression in three (one local and two pulmonary) and recurrence in five (peritoneal and pulmonary in two, and liver, pulmonary, and local recurrence in one patient each).

**Figure 1:**
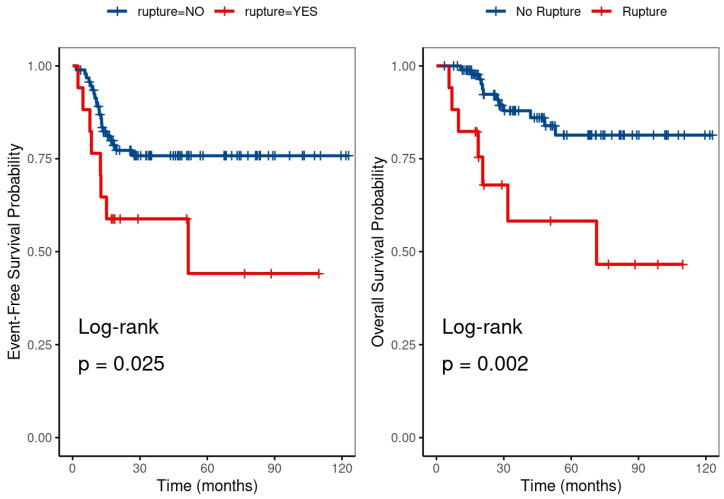
Kaplan–Meier event-free survival and overall survival curves for patients with and without ruptured Wilms tumor.

Survival analysis demonstrated significantly poorer outcomes for patients with ruptured WT. EFS at 5 years was 44.1% for the ruptured group versus 75.8% for the non-ruptured group (*p* = 0.025). OS at 5 years was 58.2% in the ruptured group compared to 81.4% in those without rupture (*p* = 0.002), Figure 1.

On multivariable Cox regression analysis, tumor rupture was found to be an independent predictor of relapse (HR 8.1; 95% CI: 1.7–39.6; *p* = 0.01), Table 2, and death (HR 17.62; 95% CI: 2.69–115.48; *p* = 0.003), Table 3.

## 4. Discussion

Previous studies have reported WT rupture rates ranging from 2% to 10%. In our cohort, rupture was observed in 15.3% of cases, which is slightly higher than reported in the literature [5,6,7]. Pre-operative tumor rupture in our series may be related to delayed presentation with a large tumor size or more aggressive tumor biology. In a report from the National Wilms Tumor Study (NWTS)-4, tumors measuring ≥10 cm in diameter were associated with a higher risk of surgical complications [9]. Similarly, the Children’s Oncology Group (COG) identified large tumor size as a significant risk factor for intraoperative tumor spillage. They reported an overall spillage rate of 11.9%, with the likelihood of intraoperative spillage being 2.18 times higher in patients whose tumors measured ≥12 cm compared with those <12 cm [7]. In our cohort, the median size of those with pre-operative rupture was 13.7 cm in comparison to 11.7 cm in those without rupture, which is statistically significant.

The majority of ruptures in our study were spontaneous and were closely associated with larger tumor dimensions and hemorrhagic necrosis. Larger, necrotic, and more rapidly growing tumors are more likely to be at risk of spontaneous rupture, reflecting a biologically more aggressive phenotype [6,7,19]. Adverse tumor biology may also contribute to tumor friability and rupture risk. Importantly, molecular markers associated with poorer prognosis—such as loss of heterozygosity (LOH) at chromosomes 1p and 16q—were not evaluated in our cohort. These biomarkers have been identified in NWTS and COG analyses as independent predictors of relapse and inferior event-free and overall survival, reflecting more aggressive tumor behavior [20,21]. The absence of molecular characterization in our series limits the ability to determine whether underlying high-risk genetic features contributed to the observed rupture rate. Integrating molecular markers, such as LOH 1p/16q, with post-treatment blastemal volume into future protocols may allow for more refined, biologically driven risk-stratification. For instance, patients with rupture but favorable molecular profiles might eventually be spared some treatment intensity, while those with both rupture and adverse markers would be prioritized for the most aggressive therapeutic arms.

Clinically, patients with ruptured WT also had the characteristic triad of pallor, abdominal distension, and pain, due to acute intraperitoneal hemorrhage, accompanied by anemia. The same presentations were described in earlier cohort clinical series and case reports [1,5,6]. In line with Gupta et al., our series also had significantly lower median hemoglobin (7.9 g/dL) in patients with rupture, reflecting the link between rupture and hemorrhagic complications [22].

The timing of elective surgery for WT is variable. The NWTS recommends primary surgical resection, followed by chemotherapy and radiotherapy as guided by pathology and staging. The SIOP Nephroblastoma Trial Group adopts the concept of neoadjuvant chemotherapy, and further therapy is determined according to pathological stage and tumor volume. Despite the fundamentally different approach, parallel trials have shown no significant differences in event-free survival or overall survival outcomes. According to current COG and SIOP staging and treatment protocols, all children with pre-operative or intra-operative tumor rupture are upstaged to stage III disease locally. This is mainly because tumor spillage increases the risk of abdominal recurrence up to 20% [6]. A recent study emphasizes that histologic findings for confirmation of either rupture or hemorrhage are a prudent, evidence-based approach to avoid over-or under-diagnosing rupture and its associated risks [10].

Clinical signs suggestive of tumor rupture, such as acute abdominal pain, a drop in hemoglobin, or recent abdominal trauma, may be absent in some patients, particularly those with minor hemorrhage or leak. Therefore, radiologic suspicion of preoperative WT rupture is essential for accurate diagnosis and appropriate management. Preoperative CT scan is a critical diagnostic tool, and our experience aligns with previous reports in the literature. Informative radiologic signs of rupture, such as peritumoral bleeding, capsular disruption, and intraperitoneal fluid accumulation beyond the cul-de-sac, were observed in most involved patients [6,8,11,23]. In particular, 65% of ruptured tumors in our cohort had peritoneal hemorrhage or nodules, consistent with the pattern described by Kalapurakal et al. [13]. Other studies, including the AREN03B2 cohort, have shown that CT has moderate specificity but relatively low sensitivity for detecting preoperative rupture, with sensitivity ranging from 54% to 70% and specificity around 88%, and substantial interobserver agreement (κ = 0.76). Among the imaging features assessed, ascites beyond the cul-de-sac was the single most predictive sign of rupture, followed by perinephric fat stranding and retroperitoneal fluid. Most imaging signs, except peritoneal implants, intratumoral hemorrhage, and subcapsular fluid, were significantly associated with rupture (*p* ≤ 0.02), whereas the attenuation of ascitic fluid did not correlate with rupture (*p* = 0.999) [6]. Such observations highlight the crucial role of contrast-enhanced CT not only in preoperative staging and operative planning but also in rupture identification. Given the significant discordance found in our study, relying on a single modality is insufficient. We advocate for a ‘highest-stage’ principle: if any one of the three pillars—preoperative imaging, surgical observation, or pathology—indicates rupture, the patient should be managed as Stage III to ensure they receive the necessary treatment intensification. Combining CT features with clinical markers may further improve rupture detection sensitivity [15].

In the SIOP protocols, definitive confirmation of tumor rupture relies on pathological findings suggestive of rupture, which is considered the diagnostic gold standard and is performed after four to six weeks of neoadjuvant chemotherapy. However, neoadjuvant chemotherapy may induce peritumoral capsule formation, which can obscure or reconstitute capsular integrity, thereby masking pre-existing rupture and leading to discordance between clinical, radiological, intraoperative, and histological findings [24]. In our cohort, the tumor capsule was reported as intact in ten patients, despite intraoperative evidence of rupture in three cases, radiological bloody ascites in two, and peritoneal nodules and hemorrhage in two others, underscoring the potential inaccuracy of pathological rupture assessment following neoadjuvant therapy. Specifically, in our cohort, while only 35.3% of ruptured cases were confirmed pathologically, 58.8% showed a ‘masking effect’ where the capsule appeared intact despite radiological or surgical evidence of breach. This indicates that pathology alone would have under-staged more than half of the ruptured cases in a SIOP-based setting. This suggests that neoadjuvant chemotherapy may facilitate the formation of a ‘pseudocapsule’. This fibro-inflammatory response can seal prior breach sites, rendering them invisible to the pathologist but not mitigating the initial peritoneal seeding that occurred at the time of rupture. A key finding is the 58.8% “masking rate,” where neoadjuvant chemotherapy appeared to repair or obscure prior breach sites, resulting in “intact” capsules on final pathology. This aligns with the UK IMPORT study, which documented a 48% false-positive rate for radiologically suspected rupture when correlated with surgical and pathological findings [25]. Similar discordance has been reported in French series [23]. Under the SIOP-UMBRELLA approach, clinicians must integrate initial diagnostic imaging, intraoperative notes, and pathology rather than relying solely on post-chemotherapy histology to avoid undertreatment and ensure appropriate intensification with doxorubicin and abdominal radiotherapy.

The treatment protocols we used in ruptured WT were considerably more intense. Nearly all rupture patients (94%) received doxorubicin-based chemotherapy, and 100% received irradiation (whole abdomen or flank), a clearly more intensive therapy compared with no tumor rupture. The COG and SIOP protocols recommended intensive multimodal treatment, including anthracycline-based chemotherapy (per COG guidelines, and guided by tumor volume in SIOP protocols), and abdominal irradiation, following tumor rupture to reduce the risk of recurrence [5,10,14]. However, intensified therapy is associated with significant long-term risks. Green et al. and others have reported that such regimens may lead to sequelae, including cardiotoxicity, infertility, and secondary malignancies [2,8,16,17]. These have to be managed with careful, individualized treatment in rupture.

In our cohort, patients with tumor rupture had a markedly poor outcome, with a 5-year EFS of 44.1% and OS of 58.2%. Tumor rupture identified a high-risk subgroup in this cohort, with markedly inferior EFS and OS. Our findings contrast with recent SIOP-RTSG data from the GPOH group, where imaging-suspected rupture did not significantly impair OS (94.3%), likely because their metastatic rate was only 22.5% compared to 41% in our cohort. This suggests that in our setting, rupture may be a surrogate for more advanced or biologically aggressive disease [10]. Our non-ruptured OS (81.4%) is comparable to other middle-income SIOP cohorts (e.g., 80.9% in a Vietnamese series) but remains below high-resource benchmarks (~90–95%) and recent Saudi data showing 95.7% OS with strict protocol adherence despite 56% advanced-stage disease [26,27]. The significant survival gap (58.2% OS in ruptured vs. 81.4% in non-ruptured) underscores that even with the intensification of therapy to Stage III protocols, the inherent biological aggression or advanced stage associated with rupture in our population presents a persistent therapeutic challenge.

The poor survival observed in ruptured WT is not solely related to treatment intensity, but rather reflects a combination of advanced stage at presentation, biologically aggressive disease, and greater metastatic and peritoneal dissemination risk at presentation. In our cohort of ruptured WT, 41% had metastatic disease at diagnosis. This high metastatic rate in our ruptured cohort suggests that rupture in our setting may serve as a clinical marker for more biologically aggressive disease. Alternatively, it may be a result of delayed diagnosis, where the tumor has had more time to both outgrow its blood supply—leading to spontaneous rupture—and spread distally to the lungs or liver.

Our multivariable analysis identified tumor rupture as an independent predictor of both relapse (HR 8.1) and mortality (HR 17.62), consistent with findings from the NWTS and COG cohorts [6,18]. Similar associations between rupture and adverse prognosis have also been reported, reinforcing the role of tumor rupture as a significant negative prognostic factor in pediatric WT [18,19].

While tumor rupture and distant metastasis were both identified as significant independent prognostic factors in our multivariable analysis, their relative clinical ‘weights’ warrant consideration. Distant metastasis typically represents a high systemic disease burden, whereas localized rupture primarily impacts regional control; however, the extremely high hazard ratio for rupture in this cohort likely reflects its role as a surrogate for highly aggressive phenotypes, given that 41% of ruptured cases also presented with synchronous metastases. Furthermore, while intraoperative spills are often considered to carry a different prognostic weight than preoperative events, the overwhelming predominance of preoperative ruptures in our series (88%) precluded a statistically powered comparison of their individual impacts.

This study should be interpreted in light of several limitations. Its retrospective, single-center design introduces potential selection bias and limits generalizability beyond similar treatment settings. The relatively small number of patients with tumor rupture restricts the statistical power of subgroup analyses and may affect the precision of outcome estimates. Specifically, the overwhelming predominance of preoperative ruptures (*n* = 15) compared to intraoperative events (*n* = 2) precluded a meaningful separate statistical evaluation of survival outcomes between these two distinct clinical scenarios. The lack of centralized radiologic review and detailed molecular or biological tumor characterization limited our ability to explore the underlying links between rupture, tumor biology, and adverse outcomes. Furthermore, while tumor rupture was a significant independent prognostic factor, the small sample size and limited number of events resulted in wide 95% confidence intervals for the hazard ratios in our multivariable models. Consequently, these HR values should be viewed as evidence of a strong directional association with inferior survival rather than precise predictive estimates.

In conclusion, the high relapse and mortality observed in patients with tumor rupture likely reflect an underlying aggressive tumor biology and advanced stage at presentation. These findings highlight the need for early detection, accurate staging, and strict adherence to risk-adapted treatment protocols to improve outcomes in this high-risk subgroup.

## Figures and Tables

**Table 1:** Characteristics of Patients and Tumor in all groups.

Variable	All Patients, Number (Percentage)	Without Rupture, Number (Percentage)	With Rupture Number (Percentage)	*p*-Value
Total Patients	111	94	17	
Age at Diagnosis Median (range) years	3.8 (0.4–15.1)	3.5 (0.4–11.3)	4.2 (2.6–15.1)	0.030
Sex Females Males	63 (57%) 48 (43%)	52 (55%) 42 (45%)	11 (65%) 6 (35%)	0.500
Laterality Left Right	63 (57%) 48 (43%)	55 (59%) 39 (41%)	8 (47%) 9 (53%)	0.400
Signs and symptoms Abdominal mass Hematuria Abdominal pain Hypertension	83 (75%) 23 (21%) 38 (35%) 13 (12%)	70 (74%) 19 (20%) 30 (32%) 9 (9.6%)	13 (76%) 4 (24%) 8 (47%) 4 (24%)	>0.900 0.800 0.200 0.100
Hemoglobin at presentation median g/dL (range)	10.1 (5–14.3)	10.4 (6.5–14.3)	7.9 (5–13.2)	<0.001
Maximum tumor diameter median (range) cm	12 (2.4–23.5)	11.7 (2.4–23.5)	13.7 (3.0–20.3)	0.001
Histologic Subtypes Favorable Blastemal predominant Focal Anaplasia Diffuse Anaplasia	80 (72%) 18 (16%) 5 (4.5%) 8 (7.2%)	69 (73%) 16 (17%) 5 (5.3%) 4 (4.3%)	11 (64.7%) 2 (11.8%) 0 (0%) 4 (23.5%)	0.070
Stage Stage I Stage II Stage III Stage IV	32 (29.1%) 25 (22.7%) 19 (17%) 35 (32%)	32 (34%) 25 (27%) 9 (9.6%) 28 (30%)	0 (0%) 0 (0%) 10 (59%) 7 (41%)	<0.001
Positive lymph nodes	21 (19%)	15 (16%)	6 (35%)	0.090
Treatment Surgery Upfront Delayed Radiation therapy Chemotherapy Two drugs Doxorubicin-containing	111 (100%) 16 (14%) 95 (86%) 46 (41%) 111 (100%) 50 (45%) 61 (55%)	94 80 (85%) 14 (15%) 29 (30.9%) 94 (100%) 49 (52%) 45 (48%)	17 2 (12%) 15 (88%) 17 (100%) 17 (100%) 1 (5.9%) 16 (94%)	>0.900 <0.001 <0.001

**Table 2:** Univariate and Multivariate cox model for event-free survival (EFS) for all patients.

Parameter		EFS Univariate		EFS Multivariate	
		*p*-Value	HR, 95% CI	*p*-Value	HR 95% HR CI
Gender	Male vs. Female	0.470	0.76 (0.36–1.61)	-	-
Age at diagnosis	Mean (3.9 year)	0.009	1.14 (1.03–1.26)	0.010	1.22 (1.04–1.42)
Laterality	Right vs. left	0.700	1.15 (0.55–2.40)	-	-
Metastasis	Yes vs. No	0.001	3.93 (1.87–8.24)	0.035	0.12 (0.02–0.87)
Rupture	Yes vs. No	0.025	2.47 (1.09–5.57)	0.010	8.10 (1.66–39.57)
LN involvement	Positive vs. negative	0.003	3.20 (1.47–6.95)	0.042	4.45 (1.05–18.79)

Abbreviations: EFS, event-free survival; HR, hazards ratio; CI, confidence interval.

**Table 3:** Univariate and Multivariate cox model for Overall survival (OS) for all patients.

Parameter		OS Univariate		OS Multivariate	
		*p*-Value	HR, 95% CI	*p*-Value	HR 95% HR CI
Gender	Male vs. Female	0.800	1.13 (0.48–2.66)	-	-
Age at diagnosis	Mean (3.9 year)	0.010	1.16 (1.04–1.34)	0.020	1.36 (1.09–1.69)
Laterality	Right vs. left	0.260	1.65 (0.7–3.88)	-	-
Metastasis	Yes vs. No	0.001	4.66 (1.93–11.26)	0.020	0.03 (0.00–0.56)
Rupture	Yes vs. no	0.003	4.02 (1.62–9.99)	0.003	17.62 (2.69–115.48)
LN involvement	Positive vs. Negative	<0.001	4.47 (2.01–11.18)	0.004	11.5 (2.21–59.69)

Abbreviations: OS, overall survival; HR, hazards ratio; CI, confidence interval.

## Data Availability

The data presented in this study are available on request from the corresponding author due to ethical restrictions.

## Abbreviations

CI	Confidence interval
CT	Computed tomography
COG	Children’s Oncology Group
3D-CRT	Three-Dimensional Conformal Radiation Therapy
EFS	Event Free Survival
HR	Hazard Ratio
IMRT	Intensity-Modulated Radiation Therapy
KHCC	King Hussein Cancer Center
MDT	Multidisciplinary Team
NWTS	National Wilms Tumor Study
OS	Overall survival
SIOP	International Society of Pediatric Oncology
WT	Wilms Tumor
